# Supplementary figures and images for: Preliminary Investigation of Schmalhausen’s Law in a Directly Transmitted Pathogen Outbreak System
Source: Viruses. 2023 Jan 22;15(2):310. doi: 10.3390/v15020310 (PMC9961160; doi:10.3390/v15020310)

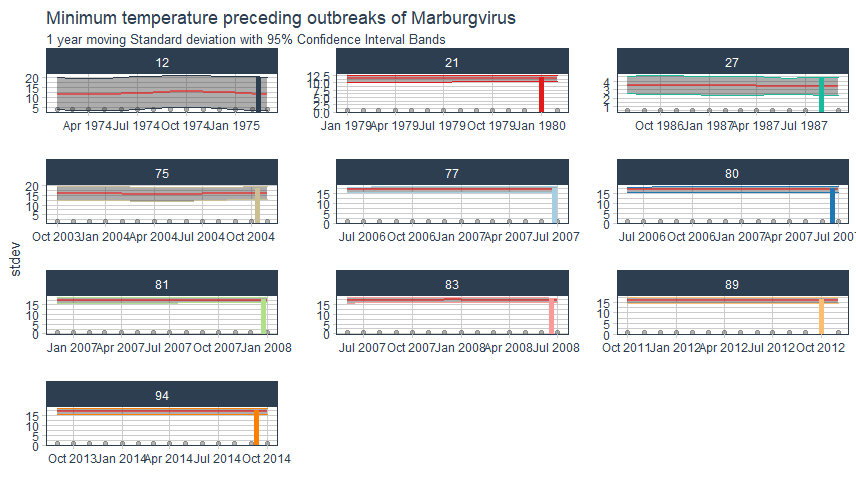

Supplement: Supplementary file 1 [file viruses-15-00310-s001.zip › Figure S1.png]

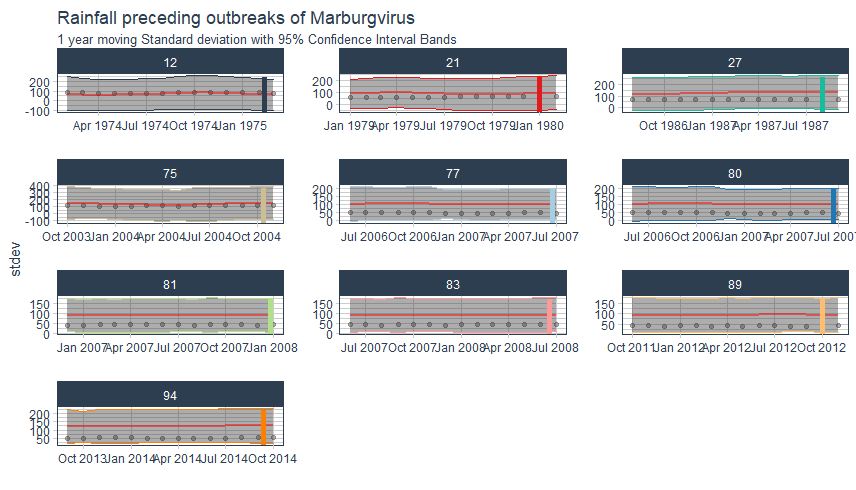

Supplement: Supplementary file 1 [file viruses-15-00310-s001.zip › Figure S2.png]
